# Supplementary material for: Single Nucleotide and Copy-Number Variants in IL4 and IL13 Are Not Associated with Asthma Susceptibility or Inflammatory Markers: A Case-Control Study in a Mexican-Mestizo Population
Source: Diagnostics (Basel). 2020 Apr 30;10(5):273. doi: 10.3390/diagnostics10050273 (PMC7277638; doi:10.3390/diagnostics10050273)
Supplement: Supplementary file 1 [file diagnostics-10-00273-s001.pdf]

Supplementary material

Table S1. Patients' clinical data.

	**AP (n=141)**
Routine blood biomarkers	
Glucose (mg/dL)	136 (115-168)
Urea (mg/dL)	27 (19-33)
Creatinine (mg/dL)	0.79 (0.67-0.92)
Arterial blood gas test	
pH	7.41 (7.39-7.44)
pCO_2	29.3 (26.3-33.0)
pO_2	53.0 (48.2-62.2)
$SatO_2$	86.0 (82.0-98.0)
HCO_3-	18.7 (16.7-20.6)

Median and interquartile range (IR 25–75) are presented for all the variables. pH: Hydrogen potential. pCO_2: partial pressure CO_2. pO2: Partial pressure Oxygen, SatO2: Oxygen saturation. HCO_3-: Bicarbonate.

Table S2. Genetic association between SNPs and clinical data.

Gene/SNP	Rhinitis (n=52) GF (%)	No rhinitis (n=80) GF (%)	p	OR	CI (95%)
		***IL13* rs20541**			
GG	22 (42.3)	33 (41.3)		1.0 (Ref)	
GA	23 (44.3)	42 (52.5)	0.33	0.81	0.39-1.72
AA	7 (13.4)	5 (6.2)		2.1	0.59-7.46
		***IL13* rs1800925**			
CC	26 (50.0)	39 (48.8)		1.0 (Ref)	
CT	21 (40.4)	31 (38.7)	0.87	1.03	0.47-2.13
TT	5 (9.6)	10 (12.5)		0.74	0.23-2.43
		***IL4* rs2070874**			
TT	16 (30.8)	24 (30.0)		1.0 (Ref)	
TC	26 (50.0)	37 (46.2)	0.82	1.05	0.47-2.36
CC	10 (19.2)	19 (23.8)		0.79	0.29-2.13
**Gene/SNP**	**Dermatitis (n=6) GF (%)**	**No dermatitis (n=126) GF (%)**	**p**	**OR**	**CI (95%)**
		***IL13* rs20541**			
GG	3 (50.0)	52 (41.3)		1.0 (Ref)	
GA	3 (50.0)	62 (49.2)	0.72	0.84	0.16-4.33
AA	0	12 (9.5)		NA	NA
		***IL13* rs1800925**			
CC	3 (50.0)	62 (49.2)		1.0 (Ref)	
CT	2 (48.3)	50 (39.7)	0.9	0.79	0.13-4.90
TT	1 (1.7)	14 (11.1)		1.31	0.14-4.52
		***IL4* rs2070874**			
TT	1 (1.7)	34 (26.9)		1.0 (Ref)	
TC	5 (98.3)	58 (46.2)	0.18	2.93	0.33-26.15
CC	0	34 (26.9)		NA	NA
**Gen/SNP**	**Allergies (n=34) GF (%)**	**No allergies (n=98) GF (%)**	**p**	**OR**	**CI (95%)**
		***IL13* rs20541**			
GG	11 (32.4)	44 (44.9)		1.0 (Ref)	
GA	20 (58.8)	45 (45.9)	0.41	1.78	0.76-4.14
AA	3 (8.8)	9 (9.2)		1.33	0.31-5.77
		***IL13* rs1800925**			
CC	19 (55.9)	46 (46.9)		1.0 (Ref)	
CT	10 (29.4)	42 (42.9)	0.32	0.54	0.22-1.29
TT	5 (14.7)	10 (10.2)		1.13	0.34-3.76
		***IL4* rs2070874**			
TT	13 (38.2)	22 (22.4)		1.0 (Ref)	
TC	15 (44.1)	48 (48.9)	0.16	0.53	0.22-1.30
CC	6 (17.7)	28 (28.6)		0.36	0.12-1.11

Genotype frequencies were compared by Fisher's exact test. For statistical significance was considered a $p < 0.05$. Ref: Genotype employed as a reference for statistical analysis. CI: Confidence intervals. OR: Odds ratio. NA: Not apply.

Table S3. Genetic association between SNPs and clinical data.

**Total leukocytes**					
**Gene/SNP**	**Normal (n=73)**	**High (n=52)**	**p**	**OR**	**CI (95%)**
	**GF (%)**	**GF (%)**			
		***IL13* rs20541**			
GG	29 (39.7)	24 (46.2)		1.0 (Ref)	
GA	39 (53.4)	22 (42.3)	0.41	1.47	0.69-3.11
AA	5 (6.9)	6 (11.5)		0.69	0.19-2.54
		***IL13* rs1800925**			
CC	35 (47.9)	26 (50.0)		1.0 (Ref)	
CT	33 (45.2)	17 (32.7)	0.08	1.57	0.71-3.49
TT	5 (6.9)	9 (17.3)		0.4	0.12-1.33
		***IL4* rs2070874**			
TT	20 (27.4)	13 (25.0)		1.0 (Ref)	
TC	35 (47.9)	24 (46.2)	0.87	0.95	0.40-2.26
CC	18 (27.7)	15 (28.8)		0.78	0.29-2.08

**Neutrophils**					
**Gene/SNP**	**High (n=90)**	**Normal (n=35)**	**p**	**OR**	**CI (95%)**
	**GF (%)**	**GF (%)**			
		***IL13* rs20541**			
GG	40 (44.4)	13 (37.1)		1.0 (Ref)	
GA	41 (45.6)	19 (54.3)	0.68	0.7	0.31-1.61
AA	9 (10.0)	3 (8.6)		0.98	0.23-4.15
		***IL13* rs1800925**			
CC	42 (46.7)	18 (51.4)		1.0 (Ref)	
CT	36 (40.0	12 (34.3)	0.7	1.29	0.55-3.02
TT	9 (10.0)	5 (14.3)		0.77	0.23-2.63
		***IL4* rs2070874**			
TT	23 (25.6)	10 (28.6)		1.0 (Ref)	
TC	41 (45.6)	18 (51.4)	0.6	0.99	0.39-2.50
CC	26 (28.9)	7 (20.0)		1.62	0.53-4.94

**Lymphocytes**					
**Gene/SNP**	**High (n=109)**	**Normal (n=16)**	**p**	**OR**	**CI (95%)**
	**GF (%)**	**GF (%)**			
		***IL13* rs20541**			
GG	47 (43.1)	6 (37.5)		1.0 (Ref)	
GA	53 (48.6)	8 (50.0)	0.82	0.85	0.27-2.61
AA	9 (8.3)	2 (12.5)		0.57	0.09-3.31
		***IL13* rs1800925**			
CC	51 (46.8)	9 (56.2)		1.0 (Ref)	
CT	43 (39.5)	4 (25.0)	0.39	1.89	0.54-6.59
TT	11 (10.1)	3 (18.8)		0.64	0.15-2.78
		***IL4* rs2070874**			
TT	30 (27.5)	3 (18.8)		1.0 (Ref)	
TC	50 (45.9)	8 (50.0)	0.73	0.62	0.15-2.53
CC	28 (25.7)	5 (31.2)		0.56	0.12-2.56

**Eosinophils**					
**Gene/SNP**	**High (n=62)**	**Normal (n=36)**	**p**	**OR**	**CI (95%)**

	GF (%)	GF (%)			
		***IL13* rs20541**			
GG	24 (38.7)	17 (47.2)		1.0 (Ref)	
GA	32 (51.6)	16 (44.4)	0.71	1.41	0.59-3.36
AA	6 (9.7)	3 (8.3)		1.41	0.31-6.49
		***IL13* rs1800925**			
CC	32 (51.6)	18 (50.0)		1.0 (Ref)	
CT	22 (35.5)	12 (33.3)	0.82	1.03	0.41-2.56
TT	6 (9.7)	5(17.7)		0.67	0.18-2.52
		***IL4* rs2070874**			
TT	16 (25.8)	9 (25.0)		1.0 (Ref)	
TC	28 (45.2)	17 (47.2)	0.98	0.92	0.33-2.55
CC	18 (29.0)	10 (27.8)		1.01	0.32-3.11
**Gene/SNP**	**Low (n=25)**	**Normal (n=36)**	**p**	**OR**	**CI (95%)**
	**GF (%)**	**GF (%)**			
		***IL13* rs20541**			
GG	11 (44.0)	17 (47.2)		1.0 (Ref)	
GA	13 (52.0)	16 (44.4)	0.89	1.25	0.43-2.60
AA	1 (4.0)	3 (8.3)		1.54	0.08-27.36
		***IL13* rs1800925**			
CC	9 (36.0)	18 (50.0)		1.0 (Ref)	
CT	13 (52.0)	12 (33.3)	0.38	2.16	0.71-6.65
TT	3 (12.0)	5(17.7)		1.2	0.23-6.19
		***IL4* rs2070874**			
TT	8 (32.0)	9 (25.0)		1.0 (Ref)	
TC	13 (52.0)	17 (47.2)	0.55	0.86	0.26-2.84
CC	4 (16.0)	10 (27.8)		0.45	0.10-2.01
**Basophils**					
**Gene/SNP**	**High (n=113)**	**Normal (n=9)**	**p**	**OR**	**CI (95%)**
	**GF (%)**	**GF (%)**			
		***IL13* rs20541**			
GG	49 (43.4)	2 (22.2)		1.0 (Ref)	
GA	55 (48.7)	6 (66.7)	0.46	0.37	0.01-1.94
AA	9 (7.9)	1 (11.1)		0.36	0.03-4.49
		***IL13* rs1800925**			
CC	56 (49.6)	3 (33.3)		1.0 (Ref)	
CT	41 (36.3)	5 (55.6)	0.54	0.43	0.09-1.94
TT	13 (11.5)	1 (11.1)		0.69	0.06-7.25
		***IL4* rs2070874**			
TT	29 (25.7)	4 (44.4)		1.0 (Ref)	
TC	54 (47.8)	4 (44.4)	0.39	1.86	0.43-7.99
CC	30 (26.5)	1 (11.1)		4.13	0.44-39.26

Genotype frequencies were compared by Fisher's exact test. For statistical significance was considered a $p < 0.05$. Ref: Genotype employed as a reference for statistical analysis. CI: Confidence intervals. OR: Odds ratio.

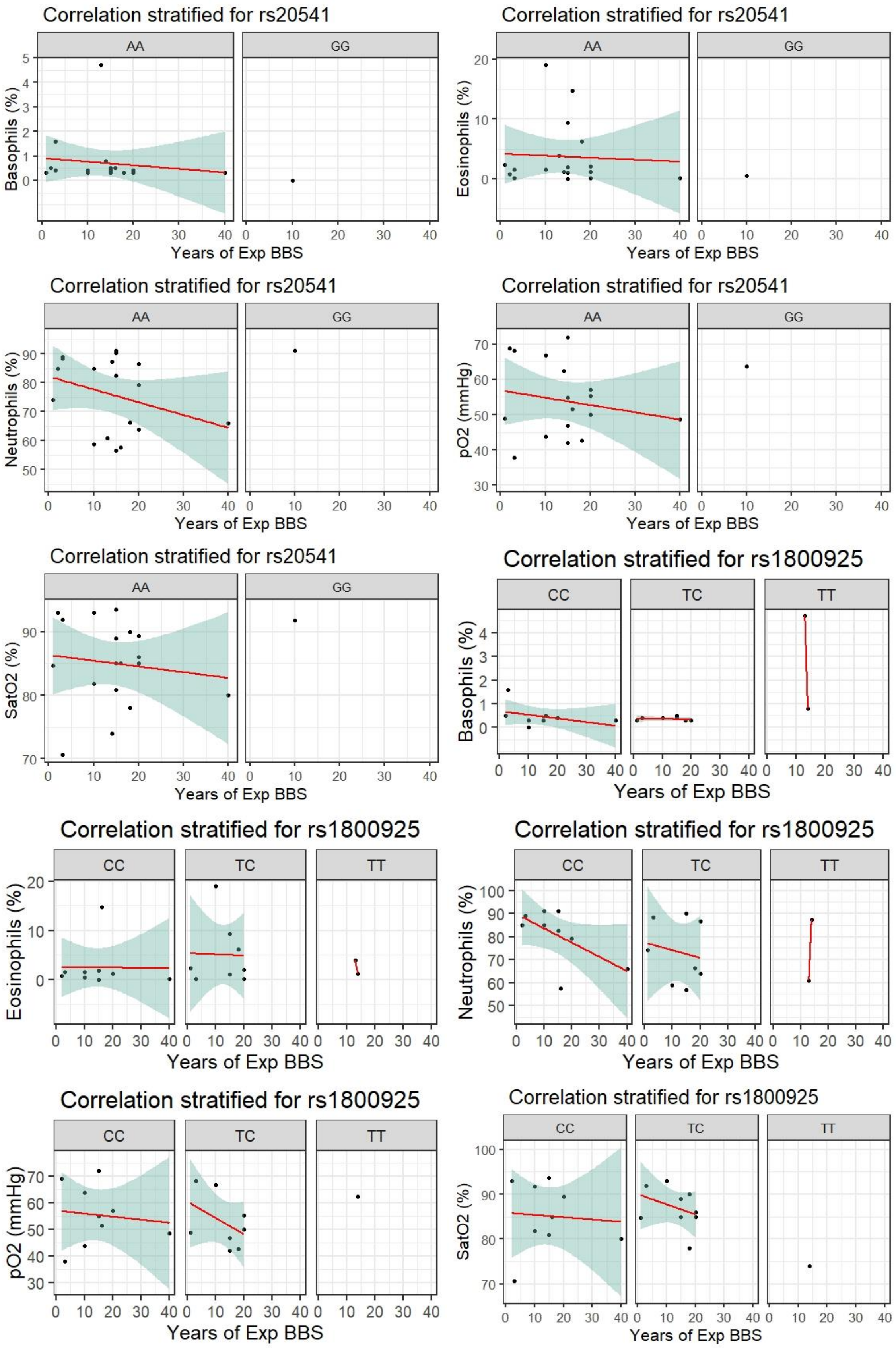
Correlation stratified for rs20541
AA
GG
Basophils (%)
Years of Exp BBS
Correlation stratified for rs20541
AA
GG
Eosinophils (%)
Years of Exp BBS
Correlation stratified for rs20541
AA
GG
Neutrophils (%)
Years of Exp BBS
Correlation stratified for rs20541
AA
GG
pO2 (mmHg)
Years of Exp BBS
Correlation stratified for rs20541
AA
GG
SatO2 (%)
Years of Exp BBS
Correlation stratified for rs1800925
CC
TC
TT
Basophils (%)
Years of Exp BBS
Correlation stratified for rs1800925
CC
TC
TT
Eosinophils (%)
Years of Exp BBS
Correlation stratified for rs1800925
CC
TC
TT
Neutrophils (%)
Years of Exp BBS
Correlation stratified for rs1800925
CC
TC
TT
pO2 (mmHg)
Years of Exp BBS
Correlation stratified for rs1800925
CC
TC
TT
SatO2 (%)
Years of Exp BBS

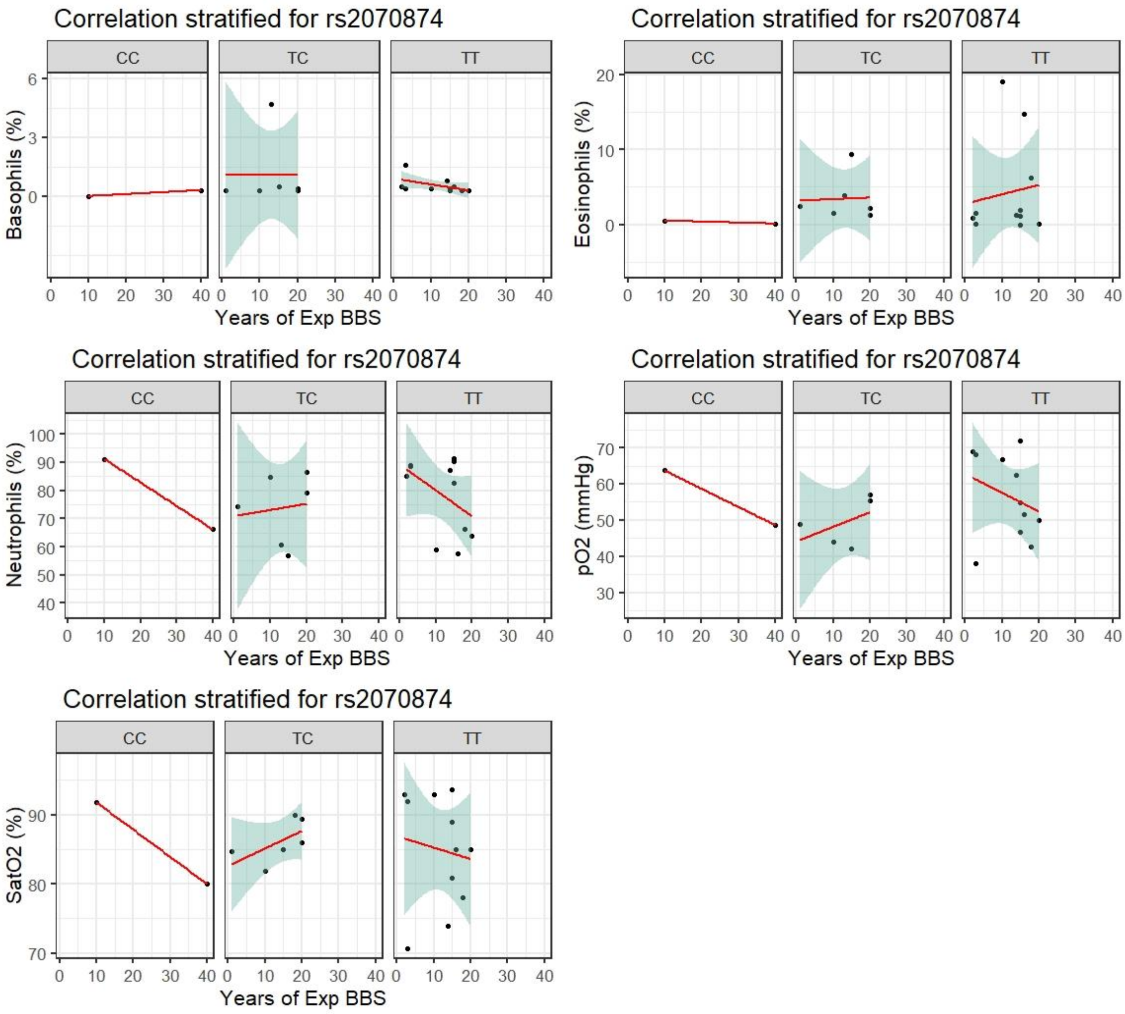

Figure S1. Correlation between cellularity and SNPs in the context of years of exposition to BBS. Blue areas show standard error (95%).

Table S4. Correlation between SNPs and cellularity/arterial blood gas values in the context of BBS.

Gene/SNP	Variable	ρ	p	CI 95%
	**Neutrophils**			
	TT	NA	NA	NA
	TC	-0.12	0.800	(-0.73 - 0.59)
	CC	-0.66	0.055	(-0.92 - 0.02)
	**Eosinophils**			
	TT	NA	NA	NA
	TC	-0.19	0.656	(-0.76 - 0.54)
	CC	-0.07	0.855	(-0.70 - 0.62)
	**Basophils**			
IL13 rs1800925	TT	NA	NA	NA
	TC	-0.37	0.368	(-0.85 - 0.45)
	CC	-0.23	0.545	(-0.78 - 0.51)
	**pO_2**			
	TT	NA	NA	NA
	TC	-0.20	0.627	(-0.76 - 0.53)
	CC	-0.06	0.881	(-0.66 - 0.59)
	**$SatO_2$**			
	TT	NA	NA	NA
	TC	-0.22	0.569	(-0.75 - 0.48)
	CC	-0.16	0.682	(-0.72 - 0.52)
	**Neutrophils**			
	GG	NA	NA	NA
	GA	NA	NA	NA
	AA	-0.25	0.318	(-0.63 - 0.23)
	**Eosinophils**			
	GG	NA	NA	NA
	GA	NA	NA	NA
	AA	-0.10	0.702	(-0.53 - 0.37)
	**Basophils**			
IL13 *rs20541*	GG	NA	NA	NA
	GA	NA	NA	NA
	AA	-0.37	0.128	(-0.72 - 0.13)
	**pO_2**			
	GG	NA	NA	NA
	GA	NA	NA	NA
	AA	-0.14	0.602	(-0.57 - 0.35)
	**$SatO_2$**			
	GG	NA	NA	NA
	GA	NA	NA	NA
	AA	-0.07	0.772	(-0.51 - 0.39)
	**Neutrophils**			

IL4 rs2070874	TT	-0.37	0.259	(-0.77 - 0.22)
	TC	0.26	0.618	(-0.61 - 0.85)
	CC	NA	NA	NA
		**Eosinophils**		
	TT	0.10	0.762	(-0.50 - 0.64)
	TC	-0.26	0.618	(-0.85 - 0.61)
	CC	NA	NA	NA
		**Basophils**		
	TT	-0.62	0.042	(-0.90 - 0.02)
	TC	0.22	0.682	(-0.64 - 0.83)
	CC	NA	NA	NA
		**pO_2**		
	TT	-0.41	0.206	(-0.80 - 0.21)
	TC	0.56	0.322	(-0.46 - 0.94)
	CC	NA	NA	NA
		**$SatO_2$**		
	TT	-0.20	0.549	(-0.70 - 0.42)
	TC	0.75	0.084	(0.00 - 0.96)
	CC	NA	NA	NA

Data obtained from Spearman's Rho correlation. pO_2: Partial pressure Oxygen, $SatO_2$: Oxygen saturation, NA: Not applicable. CI: Confidence intervals. ρ= Ro value.
